# Porencephalic cyst in adult

**DOI:** 10.4322/acr.2021.351

**Published:** 2022-01-07

**Authors:** Stefano Tambuzzi, Guendalina Gentile, Riccardo Zoja

**Affiliations:** 1 Università degli Studi di Milano, Dipartimento di Scienze Biomediche per la Salute, Sezione di Medicina Legale e delle Assicurazioni – Laboratorio di Istopatologia Forense e Microbiologia Medico Legale, Milano, Italy

**Keywords:** Autopsy, Porencephaly

Porencephalic cyst is a rare entity affecting the central nervous system, whose etiopathogenesis has not yet been clearly defined. It is a cavity within the cerebral hemisphere^1^ with smooth wall lined by gliotic or spongiotic white matter,^2^ which contains cerebrospinal fluid.^3^ The cavity usually communicates directly with the ventricular system and is often seen in territories supplied by the cerebral arteries.^2^ In other cases it may be separated by a thin layer of brain tissue and be covered externally by the arachnoid.^4^ The cavities may vary greatly in size and location, being cortical or sub-cortical, unilateral or bilateral, single or multiple.^5^ Porencephalic cysts can be classified as congenital or acquired: it has been suggested that they are caused by a disturbance of vascular supply leading to cerebral degeneration.^6^ Congenital porencephalic cysts result from intra-uterine vascular injury leading to cerebral ischemia or intra-parenchymal hemorrhage. Intra-uterine infectious injury by a virus like cytomegalovirus can also give rise to congenital porencephalic cysts.^7^ A further cause has been recognized in alcohol taken during pregnancy, as distortions in the formation of the brain with excessive plication and invaginations of the fetuses’ brain walls in alcoholic mothers have been demonstrated.^8^ Acquired porencephalic cysts are secondary to injury later in life due to trauma, surgery, ischemia, or infection.^9^ De novo or inherited heterozygous mutations in COL4A1, which encodes the type IV a1 collagen chain, have been reported in individuals with porencephaly.^6^ Finally, more recently, mutations in the tubulin alpha 1a gene (TUBA1A) have also been related to the onset of this pathological condition.^10^

From an epidemiological point of view, porencephalic cysts are described in newborns with an estimated incidence of 3.5 per 100,000 live births,^3^ and only very rarely they are reported in adults.

The symptoms that characterize porencephaly are numerous^11^ and vary with the neuronal loss that precedes the formation of cysts, or to the mass effect of the cyst itself. In the first case, cerebral palsy or convulsions, mental retardation, and learning difficulties are observed, while in the second case, the mass effect occurs with increased intracranial pressure or focal deficits attributable to localization in the involved lobe.^5^^,^ 12 ^,^ 13 In this case, epilepsy, visual, speech, and hearing impairment, rhinorrhea, or otorrhea of the cerebrospinal fluid^4^ typically occur and, last but not least, may be also observed psychiatric disorders (schizophrenia, psychosis).^14^

For the diagnosis of porencephaly, the electroencephalogram may help but the findings are not specific, and radiological techniques are necessary. A cerebral CT scan shows a hypodense intracranial cyst, with a well-defined border and central attenuation due to the cerebrospinal fluid. Mass effect on the adjacent parenchyma is not usually observed, but very large cysts can cause it locally. Brain MRI shows a well-defined, white matter-coated brain cyst with or without gliosis, with the cerebrospinal fluid signal. Fetal MRI is also helpful to differentiate this entity from extra-axial lesions such as arachnoid cysts but also rare tumors.^13^ Neurosurgical treatment is reserved for symptomatic patients, especially those suffering from drug-resistant neurological complications, such as epilepsy.^15^ In these cases, the therapeutic approaches may consist of: fenestration of the cysts in the lateral ventricle with permanent endovascular occlusion with a balloon,^16^ cortical resections,^17^ lobectomies,^18^ subtotal or total hemispherectomy,^19^ as well as a ventriculoperitoneal shunt in the case of hydrocephalus.^20^ There is no literature discussing therapy for asymptomatic porencephaly which only requires monitoring and surveillance. In some exceptional asymptomatic or pauci-symptomatic cases, the first diagnosis may only be reached upon the autopsy examination.

**Figure 1:**
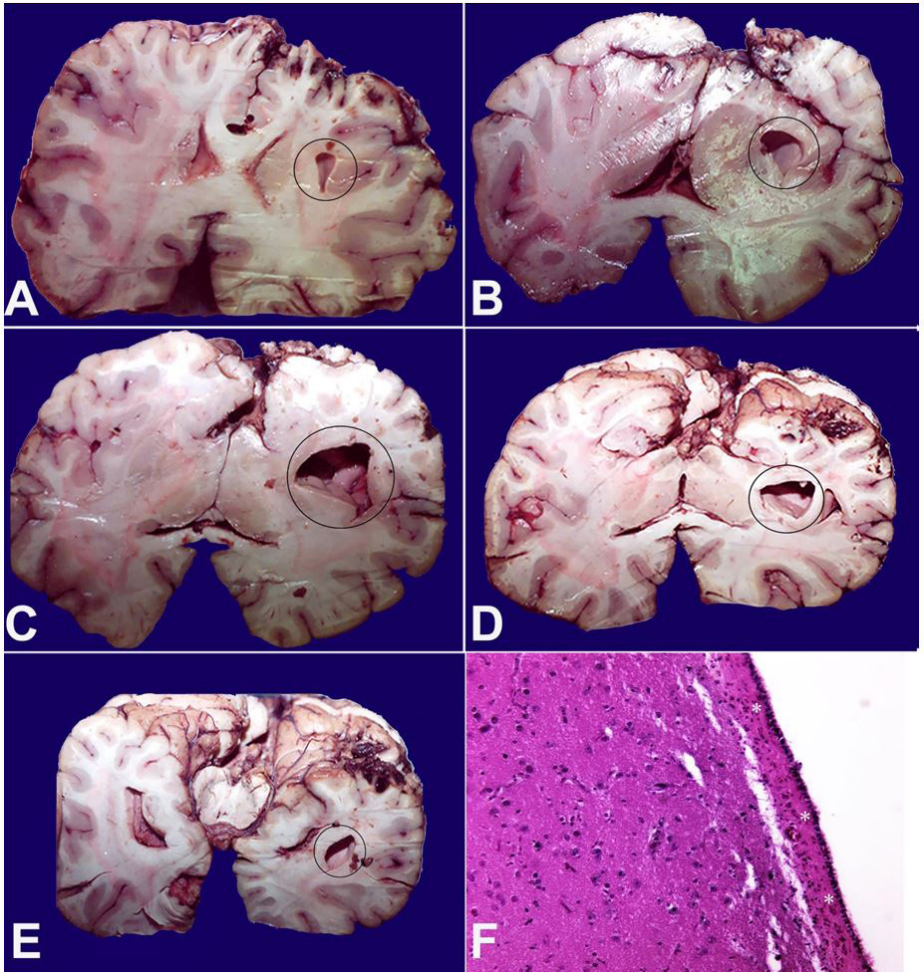
**A-E –** Macroscopic view of left craniocaudal coronal encephalic sections with evidence of the porencephalic cyst within the cerebral white matter (marked with circles); **F** – Microscopic view of the cystic cavitation at the left temporal lobe, which wall appears regular and smooth with sub ependymal lining (asterisk) (H&E, 200x).

Figures 1A-1E refer to the case of a 45-year-old man found dead in his home, prone on the bathroom floor, with no visible signs of violence on the body. To clarify the causes of death, he was subjected to a clinical autopsy. During the interview with the deceased’s relatives, it emerged that the man had been suffering for years from recurrent episodes of mild headache that he had never wanted to investigate from a medical point of view. No other pathological conditions were documented, nor the drugs or alcohol abuse.

Upon dissection of the brain (weight 1214 g [mean reference range 1366 g]), a slight asymmetry of the cerebral hemispheres was noted, with a prevalence of the left one. The leptomeninges were smooth and shiny, with no focal alterations; the vessels were marked dilated and congested. At serial coronal sections, a large cavitated formation was present in the white matter of the entire left cerebral hemisphere, in the superolateral position to the ventricle. It extended from the middle third of the frontal lobe to the middle third of the occipital lobe, reaching a maximum diameter of 2.5 cm in the left temporal area. In addition, the occipital lobe of the left cerebral hemisphere showed an abnormal supernumerary convolution. In Figure 1F, the surface of the cystic cavitation wall, which appears regular and smooth with a sub ependymal coating, can be observed.

At the end of the autopsy, the cause of death was identified in acute circulatory failure in a subject suffering from severe coronary artery disease and porencephaly. Therefore, the finding of the porencephalic cyst was incidental, and the origin of this disease was likely attributed to a congenital cause. Indeed, the man had always had good health, except only for recurrent episodes of mild headache.

We deemed this finding of interest both for the rarity of the detected cerebral anomaly and for the man’s survival until adulthood in the absence of severe symptoms. Indeed, the first diagnosis occurred only *postmortem* and the histopathological analysis confirmed the porencephalic nature of the large cerebral cavitated defect.
